# Accuracy of digital templating of uncemented total hip arthroplasty at a certified arthroplasty center: a retrospective comparative study

**DOI:** 10.1007/s00402-021-03836-w

**Published:** 2021-03-16

**Authors:** D. Dammerer, A. Keiler, S. Herrnegger, D. Putzer, S. Strasser, M. Liebensteiner

**Affiliations:** 1 Department of Orthopaedics and Traumatology, Medical University of Innsbruck, Anichstrasse 35, 6020 Innsbruck, Austria; 2 Department of Orthopaedics and Traumatology, Experimental Orthopaedics, Medical University of Innsbruck, Sonnenburgstrasse 16, 6020 Innsbruck, Austria

**Keywords:** Total hip arthroplasty, Digital templating, Experience, Accuracy, EndoCert

## Abstract

**Introduction:**

To investigate the accuracy of preoperative digital templating for total hip arthroplasty (THA) at a certified arthroplasty center (EndoCert EPZmax).

**Materials and methods:**

In a retrospective study design, we analysed 620 uncemented primary THAs for templating accuracy by comparing the preoperatively planned THA component size and the implanted size as documented by the surgeon. Templating was determined to be a) exact if the planned and the implanted component were the same size and b) accurate if they were exact ± one size. Moreover, we investigated factors that potentially influence templating accuracy: overweight and obesity (WHO criteria), sex, implant design, surgeon experience, preoperative diagnosis. Digital templating was done with MediCAD software. The Mann–Whitney *U* test and the Kruskal–Wallis test were used for statistical analysis.

**Results:**

Templating was exact in 52% of stems and 51% of cups and was accurate in 90% of the stems and 85% of the cups. Regarding the factors potentially influencing templating accuracy, the type of cup implant had a significant influence (*p* = 0.016). Moreover, greater accuracy of stem templating was achieved in female patients (*p* = 0.004). No such effect was determined for the other factors investigated.

**Conclusions:**

We conclude that preoperative 2D templating is accurate in 90% of the stems and 85% of the cups. Greater accuracy may be achieved in female patients. In addition to gender, the type of implant used may influence planning accuracy as well. Surgeon experience, BMI and preoperative diagnosis did not influence templating accuracy.

**Level of evidence:**

Level III (retrospective comparative study with prospective cohort).

## Introduction

Preoperative planning is an essential and integral part of the total hip arthroplasty (THA) procedure [1]. Digital templating of THA is well established, facilitates the determination of the correct implant size and helps restore the patient-specific physiological biomechanical conditions such as leg length, offset, center of rotation as well as lateralization [1–3]. Moreover, preoperative surgical planning improves postoperative range of motion and stability, shortens the operative time and reduces wear caused by malpositioning of the implant components [1–5].

A variety of factors that might negatively influence the accuracy of digital templating are mentioned in the current literature [4, 6–8]. Difficulties in determining the correct magnification factor for calibrating digital X-rays have been described, especially for obese patients [4, 6–8]. In addition, better results affecting the accuracy of the predicted component size using digital preoperative planning software have been shown in combination with the planner’s experience [1–4, 9–11]. According to the literature, even implant design seems to have an effect on planning accuracy [11].

There is evidence to support the issues and the extent to which digital planning matches the actual intraoperatively selected THA size [1, 7, 12, 13], but to the best of our knowledge our study is the first to investigate the accuracy of preoperative THA planning at an EndoCert EPZmax center  [14, 15]. At such a certified arthroplasty department, endoprosthetic interventions and preoperative templating are performed according to the guidelines and specifications of EndoCert [16, 17]. EndoCert is an initiative of the German Society for Orthopaedics and Orthopaedic Surgery (DGOOC) and is the world's first system for the certification of medical facilities in the field of joint replacement [14]. The EndoCert certification system is intended to ensure high-quality patient care and high patient safety in endoprosthetic procedures [16]. The process quality, interdisciplinary and constantly evolving treatment paths as well as a high level of training for all professional groups involved in the treatment are of central importance in the certification process [14, 17]. In addition, certified surgeons have to perform a predetermined number of joint replacements per year [17]. Institutions that meet the requirements can be certified as an endoprosthetic center or endoprosthetic center for maximum care [15, 17].

We, therefore, aimed to (a) determine in how many cases preoperative templating accurately matched the implant size chosen intraoperatively and (b) analyse factors that might have influenced the accuracy of preoperative digital templating in patients who underwent THA in an EndoCert max center (experience, body mass index (BMI), sex, implant design and preoperative diagnosis).

## Materials and methods

The study protocol was approved by the local ethics committee of the Medical University (No. 1150/2019) and performed in line with the principles of the Declaration of Helsinki.

We retrospectively investigated and included all patients who underwent primary THA at our department between January 2017 and August 2019. A total of 786 patients, giving a total of 843 implanted THAs, were investigated. Inclusion criteria were defined solely as the implantation of a primary uncemented total hip implant in the above-mentioned and defined period of time. Exclusion criteria were prior surgical interventions in the hip joint, previous fractures with joint involvement, cemented THA, revision surgery and intraoperative complications such as periprosthetic fractures and malalignment of the femoral stem in postoperative anterior–posterior X-rays (defined as 5° < varus or valgus). A total of 578 patients met our inclusion criteria, resulting in 620 uncemented THAs.

Socio-demographic data and patient-related factors were collected to analyse a possible influence on the preoperative planning and the intraoperatively selected component size. These factors included body mass index (BMI), preoperative diagnosis, side of the operation, cut-to-suture time, positioning of the THA (cup: inclination and anteversion; stem: 5° < varus or valgus), surgical approach as well as the planner’s experience and intraoperative and postoperative complications. We included the uncemented implants most commonly used at our department:

*Trident PSL* cup in combination with the *Accolade II* stem (both Stryker Orthopaedics, Mahwah, NJ, USA) and the *Pinnacle* cup combined with the *Corail* stem (both DePuy Synthes, Warsaw, IN, USA). The *Trident PSL* cup is 1.8 mm wider than the stated size. This is meant to achieve an interference fit at the periphery of the implant [18, 19]. The *Accolade II* has a morphologic wedge and a size-specific medial curvature [20]. The Trident-Accolade II THA was found to be a common implant combination in Germany. According to the annual report of the German Joint Replacement Registry, 1875 Trident PSL cups and 3086 Accolade II stems were implanted in Germany in 2018 [21].

The *Pinnacle* cup is a spherical cup with a single radius [1, 22]. The *Corail* stem is designed to sit in the cancellous bone. It is hydroxyapatite-coated and has trapezoidal-like proximal cross-sections to provide rotational stability [1, 23]. According to the German Joint Replacement Registry in 2018 the Pinnacle cup was the most commonly used hip cup in Germany with a total of 17,878 implantations. The Corail stem was implanted 20046 times in Germany during the same period [21].

The decision for one or the other type of cup or stem was made by the surgeon himself, who preferred to use a particular cup or stem type, and did not depend on the patient. Thus, there is no selection bias for the implanted cup or stem component.

### Preoperative radiographs and digital templating

**Fig. 1:**
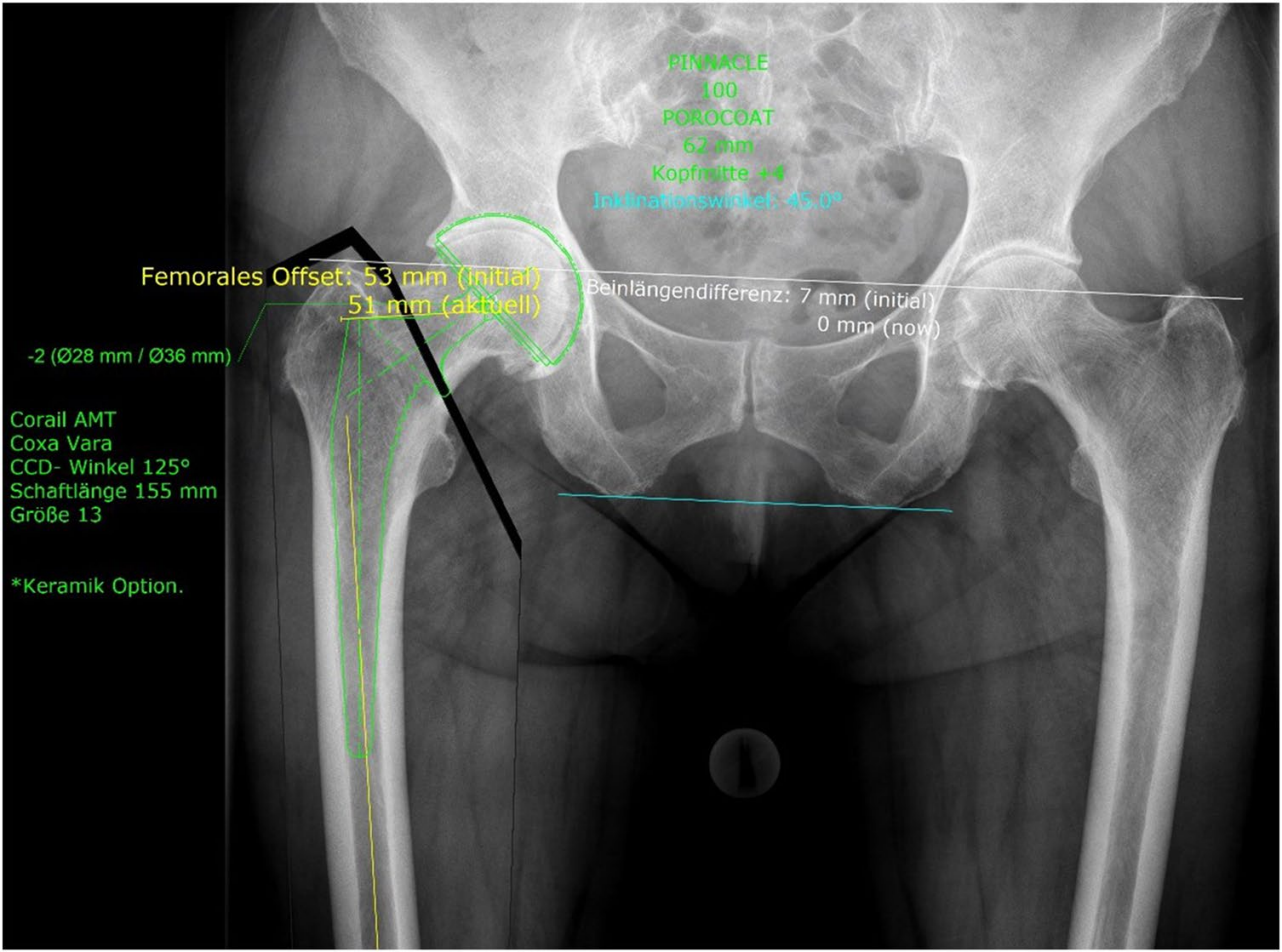
Preoperative planning. Standardised antero-posterior view of the pelvis with a correctly placed calibration marker. MediCAD software

All radiographs were taken with the same technique: anterior–posterior (AP) radiographs; patient standing in the upright position and full weight-bearing. The tube-to-film distance was standardised at 1.15 m by the Dept. of Radiology. A radiopaque ball with a diameter of 25 mm served as a size reference, which was placed in a standardised manner according to the manufacturer's recommendations: at the level of the femur shaft with symmetrical positioning of the pelvis, hip joints in a neutral position, longitudinal femur axis parallel to the image receptor plane, patella in zero position and central ray beam on femoral head center and symphysis respectively (Fig. 1) [24]. Preoperative digital templating was done with the MediCAD program (mediCAD Hectec GmbH, Altdorf/Landshut, Germany, Fig. 1). Thus, planning was performed by both specialized and non-specialized hip surgeons. Surgeons certified by EndoCert were deemed experienced and specialized hip surgeons.

We investigated the cup and the stem separately. Accuracy of preoperative templating was determined by comparing the difference between planned and implanted component sizes as documented in the surgical report. If the planned and implanted THA components were the same size this was taken as ‘exact’. A variance of + / − one size was still considered to be accurate. Deviations of more than one size were considered inaccurate [1, 25, 26].

### Statistical analysis

Statistical analysis was performed with SPSS version 26 (IBM SPSS statistics, Chicago, IL, USA). Level of significance was set at *p* < 0.05. Descriptive statistics were applied for sex, age, BMI, preoperative diagnosis and side of the operation. The influence that the planner's experience level, the component manufacturer and the patient's sex had on accuracy was analysed with the Mann–Whitney *U* test. Correlation between BMI and planning accuracy was investigated with the Kruskal–Wallis test and paired post-hoc tests.

## Results

**Fig. 2:**
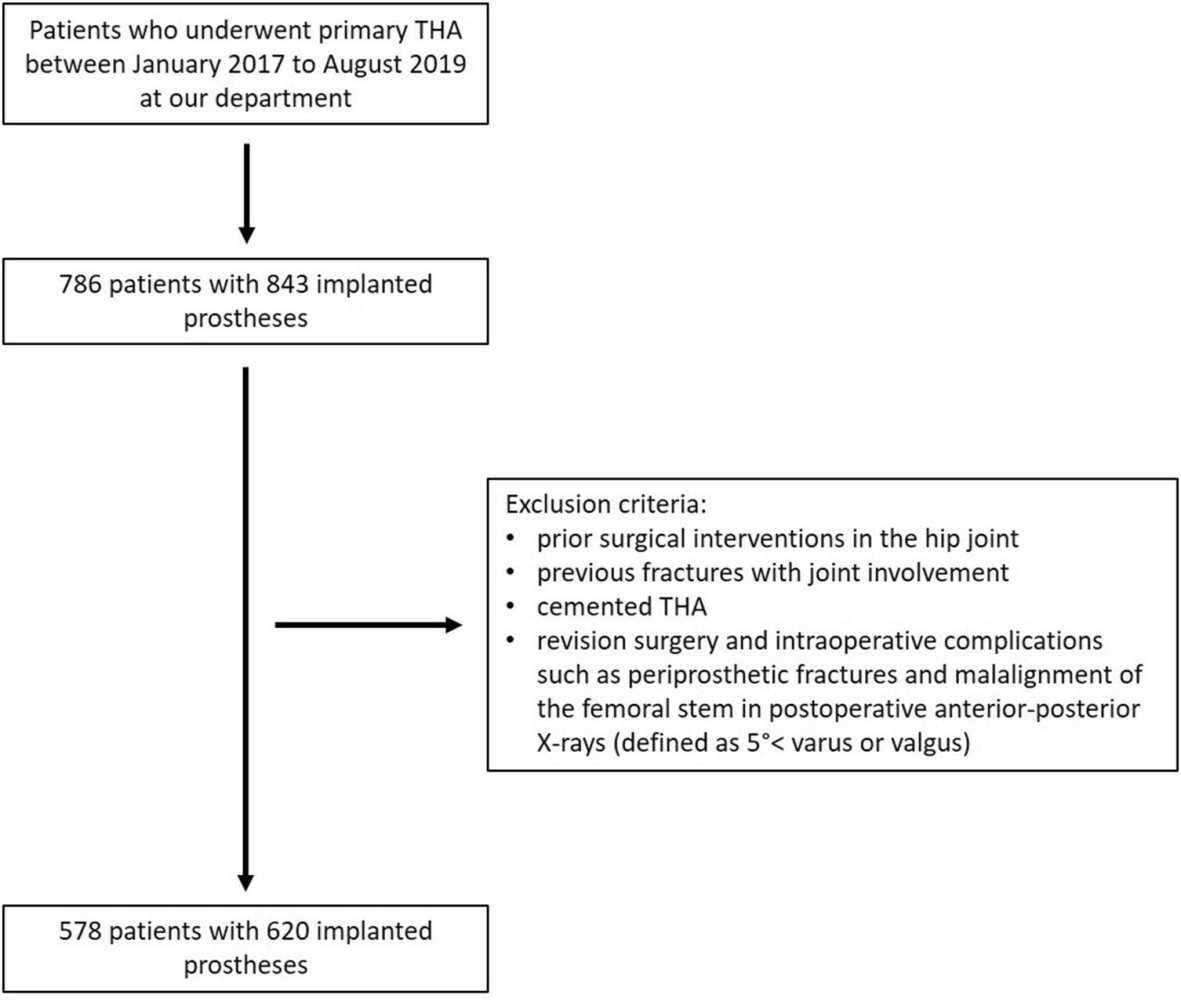
Flowchart of the study group constellation

A total of 620 (left: 277; right: 343) cementless THAs in 578 patients (female: 298; male: 280) were investigated. Mean age at surgery was 66.1 (range: 16.7–90.3) years. In 578 cases the THA was performed on one side and in 42 cases on both sides simultaneously. Mean body mass index was 26.7 (range: 16.9–59.2) kg/m^2^. According to the definition of the World Health Organization (WHO), 207 participants were under- or normal weight (BMI up to 24.9), 255 were overweight (BMI between 25 and 30) and 157 were obese (BMI from 30.1 upwards). The most common indication for THA was primary osteoarthritis in 533 out of 620 cases. In 56 cases necrosis of the femoral head, in 29 hip dysplasia and in 12 cases protrusion osteoarthritis of the hip was the indication for THA surgery. Mean cut-to-suture time was 67.7 (range 37–181) minutes. In all patients, a direct anterior approach was performed for the THA procedure [27, 28]. A Trident PSL cup and an Accolade II stem were implanted in 88% (*n* = 544), and a Pinnacle cup and Corail stem were used in 12% (*n* = 76) of the procedures. Details are given in Table 1 and Fig. 2.

**Table 1:** Demographic data of patients, distribution of BMD and primary diagnosis, mean duration of the operation and used implants

Number of patients	
Female	298
Male	280
Total	578
Operated side	
Left	277
Right	343
Total	620
Mean age in years	66.1 (16.7–90.3)
Mean BMI	26.7 (16.9–59.2)
BMI (WHO classification in kg/m^2^)	
Under- or normal weight	207
Overweight	255
Obese	157
Preoperative diagnosis	
Primary osteoarthritis	533
Protrusion osteoarthritis	12
Femoral head necrosis	56
Hip dysplasia	19
Average duration of surgery in minutes	67.7 (37–181)
Number of implanted cups	
Trident PSL	544
Pinnacle	76
Number of implanted stems	
Accolade II	544
Corail	76

### General templating accuracy

For the cups, templating was found to be exact in 51% of our cases (*n* = 315). In 34% (*n* = 208), preoperative templating differed by one size. For the stems, templating was found to be exact at 52% (*n* = 320). In 38% (*n* = 256) the preoperative templating of the stems differed by one size. In other words, in total 85% (*n* = 523) of the cups and 90% (*n* = 576) of the stems were calculated accurately (exact ± one size). Details are given in Table 2.

**Table 2:** Planning accuracy and deviation of implants in absolute values and percentage

	Cup size		Stem size	
	Occurrence	Percentage	Occurrence	Percentage
Implant size in general				
Exact match	315	51	320	52
± 1 size	208	34	256	38
± 2 sizes	66	11	35	7
± 3 sizes or more	31	5	9	3
Implant size Stryker				
Exact match	267	49	288	53
± 1 size	187	35	202	38
± 2 sizes	60	11	35	7
± 3 sizes or more	30	5	15	3
Implant size DePuy Synthes				
Exact match	48	63	32	42
± 1 size	21	28	33	43
± 2 sizes	6	8	10	13
± 3 sizes or more	1	1	1	1

	Accurate acetabular planning		Accurate femoral planning	
Planner’s experience				
EndoCert-certified surgeon	150	90	150	90
Non-EndoCert-certified surgeon	79	85	80	86
Sex				
Female	264	83	295	94
Male	259	86	260	86
Preoperative diagnosis				
Primary osteoarthritis	448	84	475	89
Protrusion osteoarthritis	11	92	12	100
Femoral head necrosis	50	93	50	93
Hip dysplasia	14	74	18	90

Deviation of one size is considered to be accurate

### Factors with potential influence on templating accuracy

A total of 544 Trident PSL cups were investigated. Of the Trident PSL cups 49% (*n* = 267) were planned exactly, and in 35% (*n* = 187) accuracy was within ± one size. Of the Pinnacle cups 63% (*n* = 48) were planned exactly and 28% (*n* = 21) accurately. Thus, the Trident PSL cup was templated accurately in 84% (*n* = 454) of our cases, and the Pinnacle cup was planned accurately in 91% (*n* = 69). The difference between the two cup types was statistically significant (*p* = 0.016). A total of 540 Accolade II stems were analysed. In 53% (*n* = 288) preoperative planning corresponded exactly to the implanted stem size. In 38% (*n* = 202) of the stems planning differed by ± one size. A total of 76 Corail stems were analysed: 42% of the stems (*n* = 32) were planned exactly and in 43% (*n* = 33) the preoperative planning was accurate. Thus, the Accolade II stem was templated accurately in 91% (*n* = 490) of our procedures, and the Corail stem was accurately planned in 86% (*n* = 65). No statistically significant difference was found in the accuracy of the preoperative planning of the Accolade II as compared to that of the Corail stem (*p* = 0.052, Table 2).

Regarding surgeon experience as a factor potentially influencing templating accuracy, it was found that certified arthroplasty surgeons achieved accuracy in 90% (*n* = 150) of cases. Non-certified surgeons accurately planned the cup in 85% (*n* = 79) and the femoral stem in 86% (*n* = 80) of procedures. No significant difference was found (cup: *p* = 0.353; stem: *p* = 0.169) (Table 2).

The planning precision in the different BMI groups, according to the definition of the WHO, showed in the group of the normal to underweight patients an accuracy of 83% (*n* = 171) for the cup and 91% (*n* = 191) for the stem. In the group of overweight participants, the cup was templated accurately in 87% (*n* = 221) and the stem in 91% (*n* = 237). In the group of obese patients in 83% (*n* = 130) the cup and in 79% (*n* = 141) the stem was planned accurately. No statistical significance was found (*p* = 0.422 for the cup; *p* = 0.216 for the stem) (Table 2).

We found a statistically significant difference in templating accuracy for the femoral stem between the two sexes (*p* = 0.004). Planning accuracy was seen to be greater in women than in men. The stem was planned accurately in 94% (*n* = 295) of implants in the female patients and in 86% (*n* = 260) in the male patients. Templating accuracy of the cup did not significantly differ between the sexes (*p* = 0.602). In 86% (*n* = 259) of the men and 83% (*n* = 264) of the women the cup component was templated accurately.

Regarding a potential effect of the type of diagnosis on templating accuracy, no statistically significant results were found (*p* = 0.176 for the cup, *p* = 0.354 for the stem). In patients with primary osteoarthritis accuracy was reached in 84% for the cup (*n* = 448) and in 89% for the stem (*n* = 475). In protrusion osteoarthritis, 92% (*n* = 11) of the cups and 100% (*n* = 12) of the stems were predicted accurately. In femoral head necrosis 93% (*n* = 50) of the cups and the stems were planned accurately. In patients with hip dysplasia templating was accurate in 74% (*n* = 14) of cups and in 90% (*n* = 18) of stems (Table 2).

## Discussion

The most important findings of the study were that templating was found to be accurate in 90% of the stems and in 85% of the cups. Regarding the investigated factors that potentially influenced templating accuracy, it was found that the type of cup implant had a significant influence (*p* = 0.016). Moreover, greater accuracy of stem templating was achieved in female patients (*p* = 0.004). For the rest of the investigated factors no such effect was determined.

When comparing our findings with those of previous research it appears that Holzer et al. analysed 632 preoperatively planned uncemented THAs, of which the cup and the stem were preoperatively determined to be within one size in 78% and in 87%, respectively [1]. In the study by Whiddon et al., planning accuracy was shown to be 78% for the acetabular and 90% for the femoral component (both within ± one implant size) [26]. Sershon et al. showed an accuracy of digital templating within two sizes of the final acetabular and femoral implants in 99.1% and in 97.1% of cases, respectively [25]. However, it may be questioned whether ± two implant sizes should still be taken as accurate. Wiese et al. showed a planning accuracy of 71% for the acetabular and 79% for the femoral component [29]. Roughly, the results of the current investigation match those of the studies mentioned above (Table 3).

**Table 3:** Overview of comparable literature

Study	Year	Number of patients	Implants cup	Implants stem	General accuracy cup (exact size ± 1 size)	General accuracy stem (exact size ± 1 size)	Software
Holzer et. al. [1]	2019	632	Allofit® Pinnacle®	Alloclassic® Corail®	78% (*n* = 494)	87% (*n* = 547)	EndoMap software® system (Siemens Medical Solutions AG, Erlangen, Germany)
Eggli et. al. [2]	1998	100	n.a	Müller® straight	90% (*n* = 90)	92% (*n* = 92)	Software developed by Maurice E. Müller Foundation (Bern, Switzerland) and by Department of Bioengineering Clemson University (South Carolina, USA)
Davila et. al. [7]	2006	36	Pinnacle®	Summit®	86% (*n* = 31)	72% (*n* = 26)	EndoMap® software system (Siemens Medical Solutions AG, Erlangen, Germany)
Gamble et. al. [12]	2010	40	Trident®	Accolade® Omnifit®	80% (*n* = 32)	85% (*n* = 34)	OrthoView® software (version 2.0CEN, Meridian Technique Ltd, Southampton, United Kingdom)
Shaarani et. al. [13]	2013	100	Trident®	Accolade®	80% (*n* = 80)	98% (*n* = 98)	OrthoView® software (version 2.0CEN, Meridian Technique Ltd, Southampton, United Kingdom)
Whiddon et. al. [26]	2011	51	Trident®	Secur-Fit Max® Accolade®	78% (*n* = 40)	90% (*n* = 46)	Impax® digital templating software (Agfa, Mortsel, Belgium)
Wiese et. al. [29]	2020	56	Pinnacle®	Summit®	71% (*n* = 40)	79% (*n* = 44)	Impax Orthopaedic Tools® software (Agfa, Mortsel, Belgium)
Carter et. al. [38]^a^	1995	74	–	Osteonics®	–	82–96% (*n* = 61–71)	n.a
Efe et. al. [39]	2011	169	EP-FIT-PLUS® Wagner®	Polar® Proxy Plus®	78% (*n* = 132)	82% (*n* = 139)	MediCAD® software (version 2.06, mediCAD Hectec GmbH, Altdorf/Landshut, Germany)

^a^Only accuracy of implanted stems was investigated. Accuracy between less ore more experienced surgeons was compared, no overall accuracy

Underlying studies, like the present work, are preoperative predictions based on two-dimensional electronic X-ray images. However, today there are numerous other options for preoperative planning in THA. Studies using CT-based three-dimensional planning show a significantly higher planning accuracy than do preoperative predictions based on two-dimensional electronic X-ray images. Sariali et al. and Osmani et al. demonstrated an accuracy of more than 95% when planning was based on three-dimensional CT images [30, 31]. The three-dimensional imaging based on CT images provides surgeons with more bone structures to assist in planning and increase accuracy [32]. Schiffner et al. were able to demonstrate the superiority of CT-based three-dimensional planning over 2D planning but emphasized that greater planning accuracy did not necessarily mean better clinical outcome [33]. Additionally, nowadays it is possible to plan automatically using CT imagination. Kagiyama et al. developed a system that is able to determine the most suitable implant by collecting data from an experienced surgeon [34]. Nevertheless, CT-based planning is still controversial. While Rübberdt et al. pointed out the greater radiation exposure in the area of the gonads, Henckel et al. argued that radiation exposure is negligible if CT scans are performed with special low-dose recordings [35, 36]. Furthermore, technical opportunities for preoperative three-dimensional imaging based on CT scans are not given in every hospital and sufficient precision can also be achieved with two-dimensional planning methods. Although the methods of CT-based three-dimensional and computer-assisted planning are manifold, the individual orthopaedist achieves the safest results with the method in which he was trained and has experience [37]. Thus, two-dimensional X-ray-based planning is still the most widely used in clinical practice. Though there is a manifold number of two-dimensional planning software, technically the majority is based on the same process. Preoperative radiographs are taken to a standard antero-posterior view of the pelvis and a calibration object of individual size is placed between the legs of the patients. The digital templating software automatically calibrates the image and template-overlays according to the known size of the marker. Most clinics have switched from planning with analogue solid templates to digital planning, and therefore the current literature clearly shows that digital two-dimensional planning is well established [4].

The presented study analysed the Trident PSL and the Pinnacle acetabular component systems and demonstrated that the Pinnacle cup achieved greater templating accuracy than did the Trident PSL cup (*p* = 0.016). When comparing the different femoral components, no significant difference was seen between the Accolade II and the Corail stem (*p* = 0.052). One possible explanation might be found in the “peripheral self-locking” system of the Trident PSL cup. The outside diameter of the cup is 1.8 mm wider than the actual reamed size. The surgeon has to take this into consideration when planning the cup size as well as intraoperatively when reaming the cup [18]. Furthermore, the differences in bone stock from patient to patient might have an influence, as surgical protocols mention that reaming should be performed with special attention to bone quality [18].

The presented study showed a tendency to predicting the acetabular component too large (30% planned too large, 19% too small), which coincides with the results reported by Wiese et al., where a tendency to estimate both components too large was proven [29].

Several studies have reported the experience of the orthopaedist who performed the planning to be a significant factor influencing the accuracy of preoperative planning. Carter et al. demonstrated a significant impact on the planning of both components, Holzer et al. only for the femoral component [1, 38]. Efe et al. and Strøm et al. showed no significant influence of the experience of the planning orthopaedic surgeon [39, 40], which is in line with the findings of the present study (*p* = 0.353 for the acetabular component; *p* = 0.169 for the femoral component). This might be due to the fact that the study population could possibly be too small since the planning surgeon could be identified in only 42% of the performed operations. However, the results show a trend, according to which specialized arthroplasty surgeons are more precise in their preoperative planning. With a larger study population, this might be a statistically significant influence.

No statistically significant impact of BMI was found on the accuracy of preoperative planning (*p* = 0.422 for the acetabular component; *p* = 0.216 for the femoral component). Similarly, Sershon et al. showed no significant effect of BMI on templating accuracy [25]. Whiddon et al. divided the patients into obese and non-obese patients (BMI ≥ / ≤ 30) and did not assess any differences in the accuracy of planning [25, 26]. In contrast, Holzer et al. showed a difference between normal- and overweight patients (BMI 18.5–24.9 for normal-weight patients or 25–29.9 for overweight patients) regarding accuracy [1].

In the presented study, significantly greater precision in preoperative planning was observed in female patients than in male patients, but only for the femoral component (*p* = 0.004) and not for the acetabular component (*p* = 0.602). Holzer et al. found no significant difference in the accuracy of preoperative planning between sexes [1].

Templating with accurate and reliable calibration markers is of the utmost importance in THA, as it has been shown that calibration errors using external calibration markers significantly influence component sizes [41]. In a recent study, Warschawski et al. compared the accuracy in the preoperative component selection of the double marker (King Mark) method, which may be more accurate than a single marker method, with the conventional metal ball method in the general population and in obese patients. However, the study found no difference between the King Mark method and the conventional metal ball method in the ability to accurately predict component sizes. In the subgroup of obese patients, the King Mark technique offered no advantage for accurately predicting component sizes [42].

Recently, Kase et al. presented a classification system to aid surgeons during their preoperative analysis, outlining the importance of considering femoral head translation during preoperative templating [43]. The authors describe a classification system to distinguish five types of architectural hip deformities, based on femoral head translation patterns, and advise surgeons to adapt their templating strategy accordingly. In a consecutive study, the authors evaluated whether mismatch between planned and real implant sizes compromises THA outcomes [44]. According to the given results, implanting a component of a different size than planned seemed not to compromise THA outcomes in terms of the Forgotten Joint Score (FJS) and Oxford Hip Score (OHS). Therefore, the authors advise that surgeons should respect their intraoperative findings when it comes to the ultimate implant size selection.

The following limitations are acknowledged. There was a lack of variability in the study population and therefore the subgroups were too small. Thus, the accuracy in planning for the different underlying diagnoses is not sufficiently comparable (osteoarthritis, dysplasia, femoral head necrosis etc.). Another limiting factor is the small amount of information available about the planning surgeon due to the retrospective character of the study. Of 620 included hip prostheses only 260 planning surgeons could be assessed. The study demonstrates a trend, according to which planning accuracy tends to increase with the experience of the planning surgeon, but no statistical significance was found (*p* = 0.353 for the cup; *p* = 0.169 for the stem). With a larger study population, there might be a relevant possibility to detect a significant difference.

## Conclusions

From our findings, we conclude that preoperative 2D templating is accurate in 90% of the stems and 85% of the cups. In female patients, greater accuracy may be achieved. In addition to gender, the type of implant used may influence planning accuracy as well. Surgeon experience, BMI and preoperative diagnosis did not influence templating accuracy.
